# Fellow-Workers and Their Doings

**Published:** 1914-05-09

**Authors:** 


### ROUND THE WORLD.

### Fellow-Workers and their Doings.

[The Editor Invites Early Information and Contributions Marked "Fellow-Workers."]

It is very satisfactory that Sir St. Clair Thomson was able to report at a recent meeting of the Medical Society of London that progress is being made in the treatment of laryngeal tuberculosis, a condition that has been the despair of practitioners. Sir St. Clair Thomson, who has had special opportunities of studying laryngeal forms of tuberculosis, in his capacity of laryngologist to the King Edward VII. Sanatorium at Midhurst, speaks highly of the effect of sanatorium conditions in ameliorating the local inflammation, and favours rest and the use of the galvano-cautery as important aids to recovery.

Dr. Cuthbert Morton, M.D. Edin., is finding time from his technical labours to further the movement in favour of revising anatomical nomenclature on an international basis, a quest in which his knowledge of German will stand him in good stead. Dr. Morton is honorary demonstrator of anatomy at Leeds University.

The vacancy on the senior staff at the Cancer Hospital, Brompton, has been filled by the election of Mr. C. W. Rowntree, F.R.C.S., the second assistant surgeon. Mr. Rowntree, who has been surgical registrar at the Middlesex Hospital, is being promoted over the head of the senior assistant surgeon, who has now been passed over for three successive vacancies on the full staff. The situation thus created must be almost unique at a leading London hospital. The well-known instance at a leading London medical school of a celebrated assistant surgeon who preferred that position to the care of beds in the hospital and refused all promotion is, of course, on quite a different footing.

Dr. Ambrose Spong, who has been appointed honorary surgeon to the Torbay Hospital, obtained his medical training both in Leeds and at the London Hospital. Qualifying M.B., Ch.B. with honours at the Victoria University, Manchester, in 1898, Dr. Spong has had a variety of experience both in resident appointments at the Leeds General Infirmary and as civil surgeon to the Akaroa Hospital in New Zealand. He became an F.R.C.S. Eng. in 1909, and M.D. of his University in 1912. Settling in practice at Torbay, he has now obtained an appointment which will give him plenty of surgical opportunity.

Dr. James Cardwell Gardner, who has just been made a J.P. for the county of Bucks, is an old St. George's and Cambridge man in practice at Amersham, where he holds several public health and other appointments. He was very well known to a past generation as a rowing Blue and successful Henley prize-winner.

Dr. Ralph Vincent, who has lately been advocating the use of unboiled milk in childhood, was largely instrumental in founding the Infants' Hospital, Westminster, where he is senior physician and director of the research laboratories. He is a pioneer in the scientific investigation of the maladies of infant life common in this country, a subject on which he is a prolific writer, and in connection with which he recently opened a discussion at the Manchester Medical Society. An old "Bart.'s" man, Dr. Vincent qualified in 1899, subsequently becoming an M.D. of Durham and M.R.C.P. Lond.

In his recent address to the obstetrical and gynæcological section of the Royal Society of Medicine on the need for research in ante-natal pathology, Dr. Amand Routh, M.D., F.R.C.P., dealt with a subject that has been very little investigated. Dr. Routh, who is one of the comparatively few investigators systematically to have attacked the difficult problems in connection with this branch, is consulting obstetric physician both to Charing Cross Hospital and the Samaritan Free Hospital.

Dr. Arthur Eastwood, M.D., Dr. Fred. Griffith, M.B., and Dr. Stanley Griffith, M.D., have just completed a report to the Local Government Board on the nature of the tuberculous infections of childhood. Dr. Eastwood and Dr. F. Griffith worked in the Board's laboratories, whilst Dr S. Griffith carried on his parallel investigations at Cambridge, and the combined report is a record of three years' very careful investigation. Their results may possibly alter our views as to the relation of the human type of tubercle bacilli to the common tubercular manifestations of infancy.

Dr. Risien Russell, whose lecture on prevalent nervous disorders at the Institute of Hygiene has attracted considerable attention, is physician to both University College Hospital and the National Hospital for Paralysis and Epilepsy in Queen Square, occupying as well the post of professor of clinical medicine at University College. Qualifying M.B., C.M. Edin. in 1886, Dr. Russell took the gold medal at the M.D. examination of the same university in 1893, and became an F.R.C.P. Lond. in 1897; he has for some years been prominently before the medical profession as a neurologist.

Mr. William Charles Bull, who has just retired from the post of aural surgeon at St. George's, has held that appointment since 1892, when he succeeded Sir William Dalby, the eminent otologist. Mr. Bull, who is an M.B. Cantab. and F.R.C.S. Eng., had a distinguished career as a student, and in early years was for a time assistant surgeon to the Belgrave Hospital for Children.

Mr. Lionel Colledge, M.B., B.C. Cantab., F.R.C.S. Eng., who is to be congratulated on his election as junior surgeon to the throat and ear department at St. George's following the above-mentioned retirement, is a student of that hospital, and occupies the position of assistant surgeon to the Throat Hospital, Golden Square, where he was formerly registrar.

Dr. W. Holder, of Hull, who has just given up the post of medical officer to one of the districts under the Sculcoates Board of Guardians, had performed duties in connection therewith for over forty years. Qualifying M.R.C.S. Eng. and L.S.A. as a Hull student in 1872, Dr. Holder remained faithful to that city, where he has held many appointments, including those of honorary surgeon to the Hull and Sculcoates Dispensary, and district medical officer under the Hull Educational Authority. In the course of a busy career Dr. Holder has constantly shown great active interest in various public subjects, and has specially written on the diseases of lead workers as well as in favour of cremation.

